# Sunday Afternoon with the Wounded

**Published:** 1924-05

**Authors:** Theodore Ruete


### SUNDAY AFTERNOON WITH THE WOUNDED.

### WORK OF THE ADAIR FUND.

BY THEODORE RUETE.

A horrid lump rose in one's throat as six hundred wounded, blinded, and shell-shocked soldiers, with nine stretcher-cases, who were cramming the stalls and boxes of the Palladium, on the afternoon of Sunday, March 30, rolled out the rousing choruses of the songs of 1914–1918, sung to them once again by seven charming ladies. Nothing, in fact, could have restrained these delegates of that pallid army, some 7,000 strong, still to be found in London's hospitals more than five years after the war. For they were enjoying themselves hugely at this, the annual effort of the organisers of the Adair Wounded Fund to provide the necessary sinews for carrying on and enlarging this truly splendid work.

### Forgotten!

The house was "sold out"—packed from floor to ceiling. And small wonder since, apart from any sentimental reasons, that marvellous man, Mr. Alan Adair, had provided a full afternoon's entertainment of talent, humour and rollicking jollity. Many of the capable artists were greeted like old friends, which, indeed, they were, often having given their services on previous occasions to hearten up these poor fellows whom the general public largely has forgotten. The spectacle presented by the Palladium that day, crammed as was the afternoon with truly moving incidents, was one long to be remembered, and a magnificent reminder to us all of what has been taking place at the Wigmore Hall every Sunday afternoon from September to June for the past three years. This actually was the eighty-fourth of these Sunday entertainments which have now been given to more than 46,000 men. This is a great achievement, especially when it is remembered that the necessary organisation is largely the work of one man, the indefatigable Mr. Leakey (Alan Adair) who was seized on a certain day of an inspiration.

### A Work of Mercy.

Week by week since then this "live wire" and his capable wife have worked to provide a few hours' distraction and amusement for the men in the London area who were broken by the war. These sufferers look forward to the entertainments with an eagerness that is wonderfully pathetic, some of them never having missed a single one. For many of them these little outings are the only spot of colour in their grey, monotonous lives; and even the drive into town furnishes a treat to those who cannot otherwise leave their hospitals. Through continual attendance at the Wigmore Hall the men have come to be at ease there. This is no charity at which they are assisting—"it is more like 'goin' home.'" The thought that somebody cares gives them courage to endure, and enables them to realise that they are not quite forgotten. Folks say the age of miracles is past, but go to Wigmore Street on a Sunday afternoon, between the hours of two and half-past four. The entertainments there are run solely upon a voluntary basis, no personal services being paid for. Everything is done for love—the truest, really the only form of charity. Variety artists of all kinds, proud to give their services, dash up in and dive out of taxis, and there is never a scarcity of performers to make up a capital programme of clean fun and blessed laughter. Hard-working drivers, too, of motor lorries and other forms of transport are there in droves, having willingly sacrificed their day of rest to steer into town, often from a considerable distance, the vehicles which have been generously lent by various firms.

### A Happy Sunday.

But first there is tea to be thought of, and the vans load up when the performance is over and deposit their human freight at various of Messrs. Lyons's cafés close at hand. Here, in an atmosphere of fellowship and camaraderie, every man gets a substantial tea, and is presented with five cigarettes, after which a move is made for the return journey, and—there is always next Sunday afternoon to look forward to. It is, indeed, the unfailing regularity of these entertainments that constitutes a large part of their value. On the one day in the week when the men feel their lot more keenly than on any other, a certain number of those who can be moved know for a surety that they will be taken out of themselves for a few jolly hours. Alan Adair considers it better consistently to brighten the lives of a certain proportion of these than to attempt an occasional entertainment of them all.

### The Cost.

To keep this sort of thing going, however, week in, week out, is no light task and one for which money is always urgently needed. There are the weekly teas to be paid for; and these, at 9d. per man—as good as one could wish for—come to fifteen guineas each week. Who wouldn't be host, or share this honour with others, to some six hundred heroes, at so paltry a price? Nine guineas insure the hall, at a nominal fee, programmes, advertisements, etc., for a concert, whose provider also becomes a host. Five guineas buy the gifts for the lucky draw, for drivers and men, and three guineas all the cigarettes, both of which items are obtained at cost. Wireless receiving-sets, complete with loud speaker, also are required for the hospitals, for the use of the men confined there, who also are entertained weekly by the Adair Wounded Fund, with cinematograph shows, concerts and lectures. An extension of this branch is contemplated, but the money for it is lacking as yet. It surely must be a reproach to many of us that Mr. Adair has not a longer waiting list upon which to draw for these expenses, and if folk only knew how happy are his weekly parties, there would be more competition to be the patrons to them. Visitors are always welcome at Wigmore Street, and a bunch of them is needed now to co-operate with the organisers of this noble work, who intend to carry it on so long as there are any wounded left. Mr. Leakey is bearing a very heavy burden indeed, but plenty of willing helpers can easily lighten it. Communications regarding the Fund should be addressed to Mr. Basil F. Leakey, at Somerset House, New Barnet.

